# Prognostic Factors and Survival Outcomes in Resectable Thoracic Soft Tissue and Bone Sarcomas

**DOI:** 10.3390/cancers18121904

**Published:** 2026-06-11

**Authors:** Gökce Yavuz, Julia Walter, Kaan Sarı, Nicole Samm, Fuad Damirov, Julia Zimmermann, Lars Lindner, Dorit Di Gioia, Rudolf Hatz, Jan M. Fertmann, Wulf Sienel, Christian P. Schneider

**Affiliations:** 1 Division of Thoracic Surgery, University Hospital LMU Munich, 81377 Munich, Germanynicole.samm@med.uni-muenchen.de (N.S.); rudolf.hatz@med.uni-muenchen.de (R.H.); jan.fertmann@med.uni-muenchen.de (J.M.F.); wulf.sienel@med.uni-muenchen.de (W.S.); christian.schneider@med.uni-muenchen.de (C.P.S.); 2 Division of Thoracic Surgery, Asklepios Lung Clinic Gauting, 82131 Gauting, Germany; f.damirov@asklepios.de (F.D.); j.zimmermann@asklepios.de (J.Z.); 3 Department of Internal Medicine III, University Hospital LMU Munich, 81377 Munich, Germany; lars.lindner@med.uni-muenchen.de (L.L.); dorit.digioia@med.uni-muenchen.de (D.D.G.)

**Keywords:** thoracic sarcoma, chest wall sarcoma, bone sarcoma, soft tissue sarcoma, grading, survival predictors

## Abstract

**Objectives:** Thoracic sarcomas are a heterogeneous group of rare mesenchymal tumors. This study aimed to identify prognostic factors for overall survival (OS) and progression-free survival (PFS) in patients undergoing resection of primary thoracic soft tissue and bone sarcomas. **Methods:** We retrospectively reviewed patients with primary intrathoracic or chest wall sarcomas who underwent surgical resection between 2005 and 2020. Eighty-four patients were included: 60 with soft tissue sarcoma and 24 with bone sarcoma. Univariate and multivariate Cox regression analyses were performed to identify prognostic factors for PFS and OS. **Results:** The most common histological subtypes were chondrosarcoma (19%) and undifferentiated pleomorphic sarcoma (17.8%). Overall, 54.8% of patients were female, and the mean age was 55.7 years (SD 17.9). Neoadjuvant and adjuvant therapies were primarily administered in intermediate- (G2) and high-grade (G3) tumors. Median OS was 28.4 months. On multivariate analysis, high tumor grade (G3 vs. G1–2) independently predicted worse PFS (HR 3.21, 95% CI 1.34–7.68; *p* = 0.01) and OS (HR 4.40, 95% CI 1.56–12.41; *p* = 0.01). Larger tumor size (HR 1.09, 95% CI 1.03–1.15; *p* = 0.001) and incomplete resection (HR 12.21, 95% CI 2.56–58.34; *p* = 0.002) were independently associated with worse OS, while lung metastases at diagnosis independently predicted worse PFS (HR 4.89, 95% CI 1.21–19.69; *p* = 0.03). **Conclusions:** Histological grade is the strongest independent predictor of survival in resected thoracic sarcoma. Surgery alone appears adequate for low-grade sarcomas, whereas multimodal treatment strategies seem particularly relevant for patients with higher-grade sarcomas.

## Simple Summary

Thoracic sarcomas are rare and heterogeneous tumors, and evidence guiding treatment strategies remains limited. This study evaluated prognostic factors in patients undergoing surgical resection for primary thoracic soft tissue and bone sarcomas. Histological grade emerged as the strongest independent predictor of both survival and recurrence, with high-grade tumors associated with significantly worse outcomes. Complete tumor resection was also critical for long-term survival, whereas incomplete resection and metastatic disease were associated with poorer outcomes. Patients with low-grade sarcomas achieved favorable results with surgery alone, while higher-grade tumors appeared to benefit from multimodal treatment strategies. These findings highlight the central role of surgery in thoracic sarcoma treatment and emphasize the importance of tumor biology and risk stratification in guiding multimodal therapy for this rare disease.

## 1. Introduction

Sarcomas are rare malignant tumors of mesenchymal origin, with an incidence of approximately 1%. They can arise from connective tissue or bone and may occur throughout the body. Thoracic sarcomas represent a heterogeneous group that includes both soft tissue and bone sarcomas, accounting for less than 5% of all thoracic malignancies [1]. Due to their rarity and wide spectrum of histological subtypes—each with distinct tumor biology, growth patterns, and responses to therapy—diagnosing and managing thoracic sarcomas present significant challenges.

Surgical resection with wide margins represents the cornerstone of curative treatment for thoracic sarcomas [2] and is frequently combined with adjuvant radiotherapy in locally aggressive tumors or following marginal resections [3,4]. Depending on tumor location and extent, surgery may involve complex chest wall or multivisceral resections, including reconstruction with prosthetic materials and muscle flaps [5]. For intermediate- and high-grade sarcomas and subtypes with a higher metastatic potential, neoadjuvant or adjuvant chemotherapy, often combined with regional hyperthermia, is commonly employed [6,7,8]. Optimal outcomes require a multimodal treatment approach in specialized centers with close interdisciplinary collaboration.

Several prognostic factors have been reported for thoracic sarcomas, primarily based on studies focusing on specific subtypes [9,10,11,12,13] or single-institution experiences [14,15,16]. Incomplete resection [13,15,17,18] and high histological grade [2,15,19,20,21] are consistently associated with inferior survival outcomes. Additional unfavorable factors include larger tumor size [2,20], metastatic disease [17,22], advanced age [21,23], and aggressive histological subtypes such as angiosarcoma [21] and undifferentiated pleomorphic sarcoma [22], whereas low-grade soft tissue sarcomas and chondrosarcomas generally show more favorable prognoses [23]. However, the rarity and heterogeneity of thoracic sarcomas have limited the statistical power and generalizability of existing studies.

The aim of this study was to evaluate long-term outcomes of patients with thoracic soft tissue and bone sarcomas following surgical treatment and to identify prognostic factors for overall survival (OS) and progression-free survival (PFS). Improved understanding of the determinants of survival may support risk stratification, treatment planning, and follow-up strategies in patients with these rare and heterogeneous malignancies.

## 2. Materials and Methods

### 2.1. Study Design and Population

Following approval by the institutional ethics committee (reference number 23-0597), we retrospectively reviewed the medical records of 84 patients who underwent surgical treatment for thoracic sarcoma between January 2005 and December 2020 at Ludwig Maximilian University Hospital, Munich, and Asklepios Lung Clinic, Gauting, Germany. The requirement for informed consent was waived due to the retrospective nature of the study. We included patients with primary thoracic sarcomas of the chest wall and primary intrathoracic sarcomas, categorizing them into two groups: thoracic bone sarcomas and thoracic soft tissue sarcomas. Patients undergoing surgery for local recurrence or for metastatic thoracic involvement from a primary sarcoma at another site were excluded.

### 2.2. Clinical Data Collection

Clinical, pathological, and treatment-related data were retrospectively collected from institutional records. Variables included age, sex, and preoperative imaging findings obtained by computed tomography (CT), magnetic resonance imaging (MRI), and positron emission tomography (PET), and preoperative lactate dehydrogenase (LDH) and alkaline phosphatase (ALP) measurements. Surgical variables included type of resection, reconstructive procedure, and resection margin status (R0–R2).

Tumor characteristics comprised location, histological subtype, tumor size, grade, Tumor–Node–Metastasis (TNM) classification, and Union for International Cancer Control (UICC) stage. Histological grading was performed according to the French Federation of Comprehensive Cancer Centers (FNCLCC) system, based on tumor differentiation, mitotic count, and tumor necrosis. Tumors were classified as low-grade (G1), intermediate-grade (G2), or high-grade (G3). Grade 4 (G4) tumours, specific to Ewing sarcoma, were included in the high-grade category. Information on neoadjuvant and adjuvant therapies, including radiotherapy, chemotherapy, and hyperthermia, was recorded.

Postoperative complications were graded according to the Clavien–Dindo classification. Thirty- and ninety-day mortality were recorded. Oncological outcomes included time to recurrence, pattern of recurrence (local or distant), overall survival, and time to death. Follow-up and mortality data were collected through a combination of direct patient contact, linkage to national or regional death registries, and contact with primary care physicians.

### 2.3. Diagnostic Tools

All patients underwent a thoracic CT scan as part of the preoperative diagnostic workup. Additionally, chest MRI was performed in 43 patients (51.2%). Preoperative PET-CT was conducted in 51 patients (60.7%) to complete staging and rule out distant metastases. In earlier years, abdominal CT (*n* = 64, 76.2%) or bone scintigraphy (*n* = 10, 11.9%) were used as alternative staging tools. A preoperative biopsy was performed in 75 patients (89.3%) to establish the diagnosis.

### 2.4. Systemic Treatment

Given the heterogeneity of the study population, various treatment protocols were applied. High-grade osteosarcomas and chondrosarcomas were treated with perioperative EURO-B.O.S.S. chemotherapy, with postoperative methotrexate added in poor responders [24]. Ewing sarcomas were managed according to the Euro-Ewing protocol with induction chemotherapy VIDE followed by adjuvant VAI or VAC. Patients with G2–G3 soft tissue sarcomas commonly received anthracycline- and ifosfamide-based regimens (AI 60/6 or AI 75/10) [7,25].

### 2.5. Statistical Analysis

Statistical analyses were performed using SPSS Version 26 (IBM Corp., Armonk, NY, USA) and RStudio version 4.0 (R Foundation for Statistical Computing, Vienna, Austria). Categorical variables were reported as counts and percentages, and the continuous variables as mean with standard deviation (SD) or median with interquartile range (IQR), depending on data distribution. Overall survival (OS) was defined as the time from surgery to death or the date of last follow-up for surviving patients. Patients lost to follow-up were censored at last contact. Progression-free survival (PFS) was defined as the time from surgery to the development of local recurrence or distant metastases. Survival curves were estimated using the Kaplan–Meier method. In addition, univariate and multivariate Cox regression analyses were applied to the entire study population using complete-case analysis to identify significant prognostic factors for PFS and OS. The proportional hazards assumption was assessed for all variables included in the Cox regression models, and no violations were identified. Effect modification by tumor type and multicollinearity among covariates were assessed using interaction terms and variance inflation factors (VIF), respectively. A *p*-value of <0.05 was considered statistically significant.

## 3. Results

### 3.1. Patient Characteristics

A total of 84 patients with primary thoracic sarcoma—60 with soft tissue and 24 with bone sarcoma—underwent resection at our institution between January 2005 and December 2020. The mean age was 55.7 years (SD 17.9), and 54.8% of patients were female (Table 1). Chondrosarcoma was the most common bone sarcoma subtype (*n* = 16, 66.7%), followed by Ewing sarcoma (*n* = 5, 20.8%) and osteosarcoma (*n* = 3, 12.5%). The most common thoracic soft tissue sarcoma subtypes were undifferentiated pleomorphic sarcoma (*n* = 15, 25%), fibrosarcoma and synovial sarcoma (*n* = 10, 16.7% each), followed by liposarcoma (*n* = 9, 15%) (Table 2). The median of maximum tumor diameter was 8.0 cm (IQR 4.9–11.5). Overall, 13 patients (15.5%) had low-grade (G1), 31 (36.9%) intermediate-grade (G2), and 38 (45.2%) high-grade (G3) sarcomas. At the time of diagnosis, 9 patients (8.3%) had lung metastases (Table 1).

### 3.2. Surgical Treatment

Wide surgical resection was performed in 38 patients (45.2%). Thirty-one patients (36.9%) underwent full-thickness chest wall or a multivisceral resection, while fifteen patients (17.9%) underwent marginal resection. Resection margins were macroscopically positive (R2) in 6 (7.1%) and microscopically positive (R1) in 15 patients (17.9%). Sixty-three patients (75%) received R0 resection (Table 1). Detailed information on tumor characteristics and intraoperative findings in patients undergoing R2 resection is summarized in Supplementary Table S1.

Depending on tumor location, partial sternum resection and partial rib resection were performed in 16 (19.0%) and 49 patients (58.3%), respectively. The maximum number of resected ribs was six, with a median of three ribs resected. Four patients (4.8%) underwent a partial scapula resection. Due to diaphragmic infiltration, 22 patients (26.2%) required partial diaphragm resection. In total, 35 patients (41.7%) underwent lung resection due to infiltration of the lung parenchyma, pulmonary vessels, bronchi, or the presence of pulmonary metastases. Pneumonectomy, segmentectomy, lobectomy, and wedge resection were performed in 1 (1.2%), 2 (2.4%), 8 (9.5%), and 28 patients (33.3%), respectively. Partial skin resection was required in 40 patients (47.6%) to allow full-thickness chest wall resection and achieve wide surgical margins due to the tumor’s proximity to the skin. Chest wall reconstruction was performed in 10 patients (11.9%) using only muscle flaps, including serratus anterior, pectoral, latissimus dorsi, or abdominal muscle. Alloplastic materials alone were used for defect coverage in 15 patients (17.9%). A combined approach incorporating both muscle flaps and alloplastic material was required in 36 patients (42.9%) (Table 3).

Postoperative complications of Clavien–Dindo grade III or higher occurred in 21 patients (25%). Grade III complications were observed in 13 patients (15.5%), most commonly seromas and wound infections requiring drainage or wound debridement with vacuum-assisted therapy. Grade IV complications, including respiratory or circulatory failure requiring intensive care unit admission, occurred in seven patients (8.3%). One patient (1.2%) died within 30 days postoperatively due to circulatory failure. In addition, two patients who underwent R2 resection died within 90 days postoperatively due to disease progression (Table 3).

### 3.3. Multimodal Treatment

Neoadjuvant therapy was administered to 33 patients, including chemotherapy in all 33 patients (39.3%), radiotherapy in 9 patients (10.7%), and regional hyperthermia in 17 patients (20.2%). Overall, 40 patients (47.6%) received adjuvant therapy, consisting of chemotherapy in 30 (35.7%) and radiotherapy or hyperthermia in 16 patients (19.0%) each (Table 1).

All patients with G1 sarcoma were treated with surgery only, except for one patient who received adjuvant radiotherapy after R1 resection for a desmoid tumor. Multimodal treatment was applied in 19 of 31 patients (61.3%) with G2 sarcomas and in 32 of 38 patients (84.2%) with G3 sarcomas. Among patients with G2 sarcomas, the most common multimodal treatment approaches were neoadjuvant or adjuvant chemotherapy combined with hyperthermia in 6 patients (19.3%) and 7 patients (22.6%), respectively. In patients with G3 sarcoma, the most frequently used multimodal regimens were neoadjuvant or adjuvant chemotherapy alone in 11 patients each (28.9%) and neoadjuvant chemotherapy combined with hyperthermia in 6 patients (15.8%). The distribution of treatment regimens according to sarcoma grade is summarized in Table 4.

### 3.4. Recurrence and Survival

Median follow-up was 52.7 months (95% CI 32.1–73.4 months). None of the patients with G1 sarcoma experienced recurrence. In contrast, recurrence occurred in 8 patients with G2 sarcoma (25.8%) and in 19 patients with G3 sarcoma (50.0%), resulting in an overall recurrence rate of 33.3% for the entire study population. Among patients with G2 sarcoma, 4 developed local recurrence, while 2 patients each developed distant metastases alone or both local recurrence and distant metastases. Of the 19 patients with G3 sarcoma, 9 experienced local recurrence, 3 developed distant metastases, and 7 developed both local recurrence and distant metastases. The most common sites of distant metastasis were the lung, liver, and bone.

Among patients who developed recurrence, the median time to recurrence was 6.2 months (IQR 4.2–23.4 months). Management of recurrence depended on its location, number, and extent and included surgical intervention, chemotherapy, regional hyperthermia, or radiotherapy, either alone or in combination. During follow-up, 12 patients (14.3%) developed a second recurrence.

**Figure 1:**
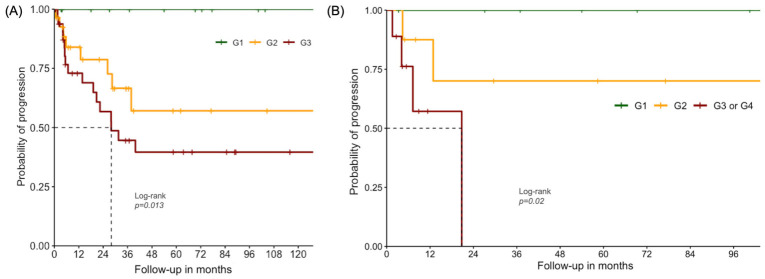
Progression-free survival for soft tissue sarcoma (**A**) and bone sarcoma (**B**) stratified by histological grade (G). Dashed lines indicate the estimated median survival.

The median progression-free survival (PFS) was 16.1 months. PFS differed significantly by tumor grade in both soft tissue and bone sarcomas (log-rank *p* = 0.013 and *p* = 0.02, respectively), with G1 tumors demonstrating the most favorable and G3 the poorest outcomes (Figure 1).

**Figure 2:**
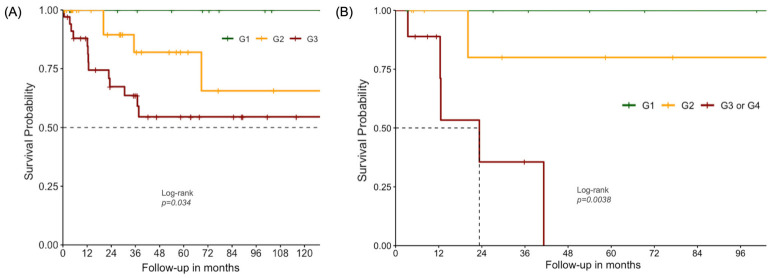
Overall survival for soft tissue sarcoma (**A**) and bone sarcoma (**B**) stratified by histological grade (G). Dashed lines indicate the estimated median survival.

The median overall survival (OS) was 29.3 months. OS likewise varied significantly by grade in both groups (log-rank *p* = 0.034 and *p* = 0.0038, respectively), with superior survival in G1 and inferior survival in G3 tumors (Figure 2).

### 3.5. Prognostic Factors for Progression-Free Survival and Overall Survival

In univariate analysis, high histological grade (G3 vs. G1–2) was significantly associated with worse PFS and OS (PFS: HR 3.16, 95% CI 1.38–7.22, *p* = 0.01; OS: HR 2.93, 95% CI 1.20–7.16, *p* = 0.02). Metastatic disease (PFS: HR 4.04, 95% CI 1.42–11.53, *p* = 0.01; OS: HR 3.87, 95% CI 1.38–10.80, *p* = 0.01), particularly lung metastases at diagnosis (PFS: HR 7.65, 95% CI 2.57–22.81, *p* = 0.0003; OS: HR 3.68, 95% CI 1.06–12.81, *p* = 0.04), and incomplete resection (R2 vs. R0; PFS: HR 6.69, 95% CI 1.82–24.67, *p* = 0.004; OS: HR 10.11, 95% CI 3.48–29.36, *p* < 0.001) were also associated with poorer outcomes. Higher preoperative alkaline phosphatase levels (HR 1.01, 95% CI 1.00–1.02, *p* = 0.02) and larger tumor diameter (HR 1.09, 95% CI 1.03–1.15, *p* = 0.002) were associated with worse OS. Age, sex, minimal resection margin, lactate dehydrogenase, and Ki-67 index were not associated with survival outcomes. No significant differences in PFS or OS were observed between bone and soft tissue sarcomas. Among soft tissue sarcomas, angiosarcoma was associated with worse PFS and OS (PFS: HR 5.60, 95% CI 1.22–25.78, *p* = 0.03; OS: HR 6.88, 95% CI 1.93–24.56, *p* = 0.003). In bone sarcomas, osteosarcoma and Ewing sarcoma were associated with worse OS compared with chondrosarcoma (HR 9.18, 95% CI 1.53–48.77, *p* = 0.02) (Table 5).

In multivariate analysis, high tumor grade remained independently associated with worse PFS and OS (G3 vs. G1–2: HR 3.21, 95% CI 1.34–7.68, *p* = 0.01; HR 4.40, 95% CI 1.56–12.41, *p* = 0.01, respectively). Larger tumor size (HR 1.09, 95% CI 1.03–1.15, *p* = 0.001) and incomplete resection (HR 12.21, 95% CI 2.56–58.34, *p* = 0.002) were independently associated with worse OS, whereas lung metastases at diagnosis were independently associated with worse PFS (HR 4.89, 95% CI 1.21–19.69, *p* = 0.03) (Table 6). No significant interaction effects between tumor type and prognostic variables were observed, indicating consistent effects across bone and soft tissue sarcoma.

## 4. Discussion

Thoracic sarcomas represent a heterogeneous group of malignancies, encompassing both soft tissue and bone sarcomas, for which surgical resection with wide margins has remained the cornerstone of multimodal treatment for decades [2]. The aim of this study was to identify the predictive factors for PFS and OS following resection of thoracic sarcomas. With 84 patients, this study represents one of the larger single-institution series of primary thoracic sarcomas reported in the literature [15,21,26,27].

Consistent with the literature, the most common histological subtypes in our cohort were chondrosarcoma (19%) and undifferentiated pleomorphic sarcoma (17.8%) [15,21,28]. High-grade sarcomas comprised 45% of cases, like previous studies reporting 40–60% of G3 tumors [2,19,23,26]. Wide surgical resection and full-thickness chest wall resection or multivisceral resection were performed in 45.2% and 36.9%, respectively. R0 resection was achieved in 75% of patients, slightly lower than recent studies reporting R0 rates of 80–85% [15,19,28,29]. This difference may be related to the larger tumor size in our cohort, with a median diameter of 8 cm and 19% of tumors exceeding 15 cm. In addition, six patients underwent R2 resection because of extensive intraoperative tumor infiltration of critical structures, including the pericardium, pleura, esophagus, and aortic adventitia, where complete resection was not technically feasible despite maximal surgical debulking. No myocardial or intracardiac involvement was identified in this cohort; however, pericardial involvement requiring partial resection was observed in a small number of cases. Our morbidity and 30-day mortality rates were 25% and 1.2%, respectively, also comparable to previously reported ranges of 13–24% and 0–1.7% [19,23].

Thoracic sarcomas are biologically heterogeneous, and treatment strategies vary according to histological subtype. Nevertheless, for certain entities such as osteosarcoma, Ewing sarcoma, and most soft tissue sarcomas, established systemic treatment protocols can be applied regardless of tumor location [7,30]. In our study cohort, protocols such as EURO-B.O.S.S. or Euro Ewing were applied for patients with osteosarcoma, dedifferentiated chondrosarcoma, or Ewing sarcoma [24,31]. Patients with soft tissue sarcoma received neoadjuvant and adjuvant chemotherapy according to the AI60/6 or AI 75/10 protocols, tailored to tumor grade and stage. Selected patients additionally underwent regional hyperthermia based on evidence supporting its potential survival benefit [32,33].

Nearly half of patients with G2–G3 sarcomas received induction therapy, predominantly chemotherapy, with a subset also treated with hyperthermia, consistent with previous reports [23,27]. Induction radiotherapy was used less frequently, reflecting its selective application in high-grade disease. Adjuvant therapy was administered in approximately half of the cohort and included combinations of chemotherapy, radiotherapy, and hyperthermia according to individual risk profiles [29,30].

Despite these multimodal approaches, local recurrence occurred in 26% of patients and distant metastases in 18%, rates comparable to or slightly lower than those reported in the literature (14–33% and 20–40%, respectively) [14,18,20,22,27]. These findings underscore the persistent risk of relapse in high-grade thoracic sarcomas and highlight that even aggressive, guideline-based treatment may not fully overcome their biological aggressiveness. Notably, our results support the integration of regional hyperthermia in selected induction and adjuvant settings, suggesting a potential contribution to improved local control and survival outcomes [32,33].

Several prognostic factors have been reported for thoracic sarcomas, including advanced age [21,23], resection margin or status [13,15,17,18], larger tumor size [2,20,21], and aggressive histological subtypes [21,22], all associated with poorer survival. In line with the literature, our multivariate analysis identified higher tumor grade as the only independent factor associated with both worse PFS and OS [2,10,15,20,21]. Additionally, larger tumor size and incomplete resection (R2) were independently associated with worse OS, supporting previous findings [17,21], whereas microscopic positive margin (R1) was not linked to worse survival, in contrast to other reports [10,15]. Metastatic disease at initial presentation, particularly lung metastases, was an unfavorable prognostic factor for both PFS and OS in univariate analysis, with lung metastases remaining independently associated with worse PFS, consistent with prior studies [17]. Histology also influenced outcomes: chondrosarcoma was associated with more favorable OS compared to Ewing sarcoma and osteosarcoma [23], while angiosarcoma showed significantly worse PFS and OS in univariate analysis, although multivariate analysis was not performed due to small sample size [21]. Notably, age was not significantly correlated with either PFS or OS in our cohort.

Our study has some limitations. Due to its retrospective nature, some patients were lost to follow-up and were censored in survival analyses, which prevented reaching the median follow-up for certain outcomes. Another limitation is the heterogeneity of the population, including both bone sarcomas and soft tissue sarcomas, resulting in small patient numbers in subgroup analyses and reduced statistical power. Furthermore, the evaluation of (neo)adjuvant treatment effects may be influenced by selection bias, as patients receiving postoperative therapy likely represent a selected subgroup with better postoperative recovery and performance status. Therefore, all results, particularly those derived from smaller subgroups, should be interpreted with caution.

Nevertheless, we identified prognostic factors associated with worse outcomes using multivariate regression analysis and provided data from a high-volume thoracic sarcoma center. Patients with G1 sarcomas generally did not receive multimodal treatment, except for one who underwent adjuvant radiotherapy following R1 resection for a desmoid tumor, and none developed recurrence. In contrast, despite neoadjuvant and adjuvant therapies, patients with G2–G3 sarcomas frequently experienced local recurrence and distant metastases. Furthermore, patients with G2–G3 sarcomas demonstrated worse PFS and OS. These findings emphasize the prognostic value of tumor grade and highlight the importance of the critical role of a multimodal treatment approach delivered in specialized centers with close interdisciplinary collaboration.

Targeted therapies, including tyrosine kinase inhibitors (pazopanib) and trabectedin, have been increasingly used in the treatment of high-grade sarcomas to improve patient outcomes [34,35]. Recently, novel therapeutic agents and immunotherapies targeting T cells or natural killer cells have been evaluated in phase I–II trials, aiming to overcome poor survival, achieve disease stabilization in metastatic sarcomas, and provide options for patients with recurrent or treatment-resistant disease [36]. Further multicenter studies are needed to evaluate treatment strategies across diverse soft tissue and bone sarcoma subgroups and to assess the efficacy of these emerging systemic therapies in phase II and III trials.

Our findings may have practical implications for the management of thoracic sarcomas. Tumor grade stratifies patients into those more likely to benefit from multimodal management versus those suitable for upfront resection, with G2–G3 tumors more frequently prompting multidisciplinary consideration of neoadjuvant systemic treatment prior to surgery. Tumor size and extent, particularly when in contact with adjacent structures, directly influence operative strategy and the anticipated complexity of chest wall resection and reconstruction. In patients with suspected pericardial or cardiac involvement, echocardiography and cross-sectional imaging may provide valuable information for preoperative assessment and surgical planning. Incomplete resection is associated with poorer outcomes, underscoring the need for careful preoperative assessment of resectability and prioritization of margin-negative resection, which in selected cases may favor a neoadjuvant approach to improve operability and local disease control. These considerations also highlight the importance of early involvement of plastic and reconstructive surgery in complex chest wall cases. These findings support risk-adapted surveillance strategies, with intensified follow-up in patients with high-grade tumors, large tumor size, or incomplete resection.

## 5. Conclusions

Tumor grade remains the key prognostic factor in thoracic sarcomas, with surgery serving as the cornerstone of multimodal treatment. Complete surgical resection was strongly associated with improved survival, whereas larger tumor size and metastatic disease predicted poorer outcomes. Patients with low-grade sarcomas demonstrated excellent outcomes with surgery alone, while intermediate- and high-grade sarcomas frequently recurred despite multimodal therapy, highlighting the aggressive biology of these tumors.

Improving outcomes in high-grade thoracic sarcomas will require continued advances in systemic therapies and multidisciplinary treatment strategies delivered in specialized high-volume centers. Further multicenter studies are needed to optimize risk-adapted treatment approaches and evaluate emerging targeted and immunotherapeutic options for this challenging disease.

## Figures and Tables

**Table 1:** Patient characteristics.

Variables	Total (*n* = 84)
age in years, mean ± SD	55.7 ± 17.9
maximum tumor diameter in cm, median (IQR)	8.0 (4.9–11.5)
sex, *n* (%)	
female	46 (54.8%)
male	38 (45.2%)
tumor diameter in cm, *n* (%)	
<5 cm	21 (25.0%)
5–10 cm	34 (40.5%)
10–15 cm	13 (15.5%)
>15 cm	16 (19.0%)
resection type, *n* (%)	
full thickness or multivisceral	31 (36.9%)
wide	38 (45.2%)
marginal	15 (17.9%)
UICC Stage, *n* (%)	
I	17 (20.2%)
II	31 (36.9%)
III	27 (32.1%)
IV	5 (6.0%)
unknown	4 (4.8%)
lung metastases at time of diagnosis, *n* (%)	7 (8.3%)
FNCLCC, *n* (%)	
low grade—G1	13 (15.5%)
intermediate grade—G2	31 (36.9%)
high grade—G3	38 (45.2%)
unknown	2 (2.4%)
resection status, *n* (%)	
R0	63 (75.0%)
R1	15 (17.9%)
R2	6 (7.1%)
neoadjuvant therapy, *n* (%)	33 (39.3%)
chemotherapy	33 (39.3%)
radiotherapy	9 (10.7%)
hyperthermia	17 (20.2%)
adjuvant therapy, *n* (%)	40 (47.6%)
chemotherapy	30 (35.7%)
radiotherapy	16 (19.0%)
hyperthermia	16 (19.0%)

**Table 2:** Histological subtypes of thoracic soft tissue and bone sarcomas.

Histological Subtype	*n*	%
Soft tissue sarcoma	60	
Undifferentiated pleomorphic sarcoma	15	25.0
Fibrosarcoma/Myxofibrosarcoma	10	16.7
Synovial sarcoma	10	16.7
Liposarcoma	9	15.0
Angiosarcoma	3	5.0
Desmoid tumor	3	5.0
Dermatofibrosarcoma protuberans	3	5.0
Leiomyosarcoma	2	3.3
Rhabdomyosarcoma	2	3.3
Others	3	5.0
Bone sarcoma	24	
Chondrosarcoma	16	66.7
Ewing Sarcoma	5	20.8
Osteosarcoma	3	12.5

**Table 3:** Surgical treatment and postoperative complications.

Intrathoracic Resection	*n*	%
Wedge resection	28	33.3
Segmentectomy	2	2.4
Lobectomy	8	9.5
Pneumonectomy	1	1.2
Diaphragm	22	26.2
Chest wall resection		
Partial rib resection	49	58.3
Partial sternum resection	16	19.0
Partial skin resection	36	42.9
Scapula resection	4	4.8
Reconstruction		
Alloplastic material only	15	17.9
Muscle flap only	10	11.9
Both	36	42.9
Complications		
Clavien–Dindo Classification		
IIIa + b	13	15.5
IV	7	8.3
30-day-mortality	1	1.2
90-day-mortality	3	3.6

**Table 4:** Multimodal treatment by tumor grade.

	Number of Patients		
Multimodal Treatment	G1 (*n* = 13)	G2 (*n* = 31)	G3 (*n* = 38)
surgery alone	12	12	6
neoadjuvant CTX + surgery	0	1	11
neoadjuvant CTX + RT + surgery	0	1	3
neoadjuvant CTX + RHT + surgery	0	6	6
neoadjuvant CTX + RT + RHT +surgery	0	3	2
surgery + adjuvant CTX	0	3	11
surgery + adjuvant RT	1	4	4
surgery + adjuvant CTX + RT	0	1	1
surgery + adjuvant CTX + RHT	0	7	3
surgery + adjuvant CTX + RT + RHT	0	1	4

Abbreviations: CTX, chemotherapy; G, grading; RHT, regional hyperthermia; RT, radiotherapy.

**Table 5:** Univariate Cox regression analyses of prognostic factors for progression-free survival (PFS) and overall survival (OS).

	PFS				OS			
	HR	CI Low	CI Up	*p*-Value	HR	CI Low	CI Up	*p*-Value
age in years	0.99	0.97	1.01	0.28	0.99	0.96	1.01	0.33
female vs. male	0.98	0.47	2.07	0.97	0.79	0.35	1.83	0.59
LDH	1.00	1.00	1.01	0.28	1.00	1.00	1.01	0.25
ALP	1.00	0.99	1.01	0.96	1.01	1.00	1.02	**0.02**
Ki-67-Index %	0.55	0.05	5.63	0.62	0.61	0.03	12.19	0.74
tumor size and extent of disease								
maximum tumor diameter	1.04	0.97	1.10	0.29	1.09	1.03	1.15	**0.002**
M1 vs. M0	4.04	1.42	11.53	**0.01**	3.87	1.38	10.80	**0.01**
lung metastases at diagnosis	7.65	2.57	22.81	**0.0003**	3.68	1.06	12.81	**0.04**
histological grading and subtypes								
G3 vs. G1 or G2	3.16	1.38	7.22	**0.01**	2.93	1.20	7.16	**0.02**
bone vs. soft tissue sarcoma	1.48	0.60	3.64	0.40	0.99	0.39	2.53	0.98
ewing&osteo- vs. chondrosarcoma	3.87	0.71	21.12	0.12	8.63	1.53	48.77	**0.01**
angiosarcoma vs. other STS	5.60	1.22	25.78	**0.03**	6.88	1.93	24.56	**0.003**
resection margins								
R1 vs. R0	2.30	0.95	5.56	0.06	2.01	0.71	5.71	0.19
R2 vs. R0	6.69	1.82	24.67	**0.004**	10.11	3.48	29.36	**<0.0001**
minimal resection distance	0.42	0.15	1.17	0.10	1.06	0.48	2.33	0.89

Abbreviations: ALP, alkaline phosphatase; CI, confidence interval; G, grading; HR, hazard ratio; LDH, Lactate Dehydrogenase; M, metastases (TNM classification); OS, overall survival; PFS, progression-free survival; R, resection margin status; STS, soft tissue sarcoma. Statistically significant *p*-values (*p* < 0.05) are shown in bold.

**Table 6:** Multivariate Cox regression analyses of prognostic factors for progression-free survival (PFS) and overall survival (OS).

	PFS				OS			
	HR	CI Low	CI Up	*p*-Value	HR	CI Low	CI Up	*p*-Value
maximum tumor diameter	1.06	0.99	1.13	0.11	1.09	1.03	1.15	**0.001**
lung metastases vs. no lung metastases at diagnosis	4.89	1.21	19.69	**0.03**	0.56	0.08	3.84	0.55
G3 vs. G1–2	3.21	1.34	7.68	**0.01**	4.40	1.56	12.41	**0.01**
R1 vs. R0	2.12	0.85	5.28	0.11	1.70	0.59	4.87	0.32
R2 vs. R0	1.78	0.36	8.82	0.48	12.21	2.56	58.34	**0.002**

Abbreviations: CI, confidence interval; G, grading; HR, hazard ratio; OS, overall survival; PFS, progression-free survival; R, resection margin status. Statistically significant *p*-values (*p* < 0.05) are shown in bold.

## Data Availability

The data presented in this study are available on request from the corresponding author. The data are not publicly available due to ethical restrictions.

## Supplementary Materials

The following supporting information can be downloaded at: https://www.mdpi.com/article/10.3390/cancers18121904/s1, Table S1: Tumor Characteristics and Intraoperative Findings in Patients with Incomplete (R2) Resection.

## Abbreviations

The following abbreviations are used in this manuscript:

ALP	Alkaline phosphatase
CI	Confidence interval
CT	Computed tomography
CTX	Chemotherapy
DFS	Disease-free survival
G	Grading
HR	Hazard ratio
IQR	Interquartile range
LDH	Lactate dehydrogenase
MRI	Magnetic resonance imaging
OS	Overall survival
PET	Positron emissions tomography
PFS	Progression-free survival
SD	Standard deviation
STS	Soft tissue sarcoma
R	Resection status
RHT	Regional hyperthermia
RT	Radiotherapy

## References

[1] Gladish G.W., Sabloff B.M., Munden R.F., Truong M.T., Erasmus J.J., Chasen M.H. (2002). Primary thoracic sarcomas. Radiographics.

[2] Gross J.L., Younes R.N., Haddad F.J., Deheinzelin D., Pinto C.A., Costa M.L. (2005). Soft-tissue sarcomas of the chest wall: Prognostic factors. Chest.

[3] Lev D., Kotilingam D., Wei C., Ballo M.T., Zagars G.K., Pisters P.W., Lazar A.A., Patel S.R., Benjamin R.S., Pollock R.E. (2007). Optimizing treatment of desmoid tumors. J. Clin. Oncol..

[4] Engelhardt K.E., DeCamp M.M., Yang A.D., Bilimoria K.Y., Odell D.D. (2018). Treatment approaches and outcomes for primary mediastinal sarcoma: Analysis of 976 patients. Ann. Thorac. Surg..

[5] Gonfiotti A., Santini P.F., Campanacci D., Innocenti M., Ferrarello S., Caldarella A., Janni A. (2010). Malignant primary chest-wall tumours: Techniques of reconstruction and survival. Eur. J. Cardiothorac. Surg..

[6] Gronchi A., Palmerini E., Quagliuolo V., Martin-Broto J., Lopez Pousa A., Grignani G., Brunello A., Blay J.Y., Tendero O., Diaz Beveridge R. (2020). Neoadjuvant chemotherapy in high-risk soft tissue sarcomas: Final results of a randomized trial from italian (isg), spanish (geis), french (fsg), and polish (psg) sarcoma groups. J. Clin. Oncol..

[7] Gronchi A., Miah A.B., Dei Tos A.P., Abecassis N., Bajpai J., Bauer S., Biagini R., Bielack S., Blay J.Y., Bolle S. (2021). Soft tissue and visceral sarcomas: Esmo-euracan-genturis clinical practice guidelines for diagnosis, treatment and follow-up. Ann. Oncol..

[8] Woll P.J., Reichardt P., Le Cesne A., Bonvalot S., Azzarelli A., Hoekstra H.J., Leahy M., Van Coevorden F., Verweij J., Hogendoorn P.C. (2012). Adjuvant chemotherapy with doxorubicin, ifosfamide, and lenograstim for resected soft-tissue sarcoma (eortc 62931): A multicentre randomised controlled trial. Lancet Oncol..

[9] Lenze U., Angelini A., Pohlig F., Knebel C., Trovarelli G., Berizzi A., Mavrogenis A.F., Theisen J., VON Eisenhart-Rothe R., Ruggieri P. (2020). Chondrosarcoma of the chest wall: A review of 53 cases from two institutions. Anticancer. Res..

[10] Marulli G., Duranti L., Cardillo G., Luzzi L., Carbone L., Gotti G., Perissinotto E., Rea F., Pastorino U. (2014). Primary chest wall chondrosarcomas: Results of surgical resection and analysis of prognostic factors. Eur. J. Cardiothorac. Surg..

[11] Widhe B., Bauer H.C., Scandinavian Sarcoma G. (2009). Surgical treatment is decisive for outcome in chondrosarcoma of the chest wall: A population-based scandinavian sarcoma group study of 106 patients. J. Thorac. Cardiovasc. Surg..

[12] Abbas A.E., Deschamps C., Cassivi S.D., Nichols F.C., Allen M.S., Schleck C.D., Pairolero P.C. (2004). Chest-wall desmoid tumors: Results of surgical intervention. Ann. Thorac. Surg..

[13] Mullen J.T., Delaney T.F., Kobayashi W.K., Szymonifka J., Yeap B.Y., Chen Y.L., Rosenberg A.E., Harmon D.C., Choy E., Yoon S.S. (2012). Desmoid tumor: Analysis of prognostic factors and outcomes in a surgical series. Ann. Surg. Oncol..

[14] Crowley T.P., Atkinson K., Bayliss C.D., Barnard S., Milner R.H., Ragbir M. (2020). The surgical management of sarcomas of the chest wall: A 13-year single institution experience. J. Plast. Reconstr. Aesthet. Surg..

[15] Soerensen T.R., Raedkjaer M., Jorgensen P.H., Hoejsgaard A., Safwat A., Baad-Hansen T. (2019). Soft tissue sarcomas of the thoracic wall: More prone to higher mortality, and local recurrence-a single institution long-term follow-up study. Int. J. Surg. Oncol..

[16] Wald O., Islam I., Amit K., Ehud R., Eldad E., Omer O., Aviad Z., Moshe S.O., Uzi I. (2020). 11-year experience with chest wall resection and reconstruction for primary chest wall sarcomas. J. Cardiothorac. Surg..

[17] Burt M., Fulton M., Wessner-Dunlap S., Karpeh M., Huvos A.G., Bains M.S., Martini N., McCormack P.M., Rusch V.W., Ginsberg R.J. (1992). Primary bony and cartilaginous sarcomas of chest wall: Results of therapy. Ann. Thorac. Surg..

[18] Sarvan M., Etienne H., Bankel L., Brown M.L., Schneiter D., Opitz I. (2023). Outcome analysis of treatment modalities for thoracic sarcomas. Cancers.

[19] van Geel A.N., Wouters M.W., Lans T.E., Schmitz P.I., Verhoef C. (2011). Chest wall resection for adult soft tissue sarcomas and chondrosarcomas: Analysis of prognostic factors. World J. Surg..

[20] McMillan R.R., Sima C.S., Moraco N.H., Rusch V.W., Huang J. (2013). Recurrence patterns after resection of soft tissue sarcomas of the chest wall. Ann. Thorac. Surg..

[21] Harati K., Kolbenschlag J., Bohm J., Niggemann H., Joneidi-Jafari H., Stricker I., Lehnhardt M., Daigeler A. (2018). Long-term outcomes of patients with soft tissue sarcoma of the chest wall: Analysis of the prognostic significance of microscopic margins. Oncol. Lett..

[22] Thakur S., Choong E., Balasooriya A., Spelman T., Wright G., Choong P. (2022). Surgical resection of chest wall sarcomas: An analysis of survival and predictors of outcome at an australian multidisciplinary sarcoma service. ANZ J. Surg..

[23] Kachroo P., Pak P.S., Sandha H.S., Lee C., Elashoff D., Nelson S.D., Chmielowski B., Selch M.T., Cameron R.B., Holmes E.C. (2012). Single-institution, multidisciplinary experience with surgical resection of primary chest wall sarcomas. J. Thorac. Oncol..

[24] Ferrari S., Bielack S.S., Smeland S., Longhi A., Egerer G., Sundby Hall K., Donati D., Kevric M., Brosjo O., Comandone A. (2018). EURO-B.O.S.S.: A european study on chemotherapy in bone-sarcoma patients aged over 40: Outcome in primary high-grade osteosarcoma. Tumori.

[25] Casali P.G., Bielack S., Abecassis N., Aro H.T., Bauer S., Biagini R., Bonvalot S., Boukovinas I., Bovee J., Brennan B. (2018). Bone sarcomas: Esmo-paedcan-euracan clinical practice guidelines for diagnosis, treatment and follow-up. Ann. Oncol..

[26] Budacan A.M., Patel A.J., Babu P., Khalil H., Vaiyapuri S., Parry M., Kalkat M.S. (2025). Chest wall resection and reconstruction for primary chest wall sarcomas: Analysis of survival, predictors of outcome, and long-term functional status. J. Thorac. Cardiovasc. Surg..

[27] Shewale J.B., Mitchell K.G., Nelson D.B., Conley A.P., Rice D.C., Antonoff M.B., Hofstetter W.L., Walsh G.L., Swisher S.G., Roth J.A. (2018). Predictors of survival after resection of primary sarcomas of the chest wall-a large, single-institution series. J. Surg. Oncol..

[28] Park I., Shin S., Kim H.K., Choi Y.S., Kim J., Zo J.I., Shim Y.M., Cho J.H. (2019). Primary chest wall sarcoma: Surgical outcomes and prognostic factors. Korean J. Thorac. Cardiovasc. Surg..

[29] Friesenbichler J., Leithner A., Maurer-Ertl W., Szkandera J., Sadoghi P., Frings A., Maier A., Andreou D., Windhager R., Tunn P.U. (2014). Surgical therapy of primary malignant bone tumours and soft tissue sarcomas of the chest wall: A two-institutional experience. Int. Orthop..

[30] Strauss S.J., Frezza A.M., Abecassis N., Bajpai J., Bauer S., Biagini R., Bielack S., Blay J.Y., Bolle S., Bonvalot S. (2021). Bone sarcomas: Esmo-euracan-genturis-ern paedcan clinical practice guideline for diagnosis, treatment and follow-up. Ann. Oncol..

[31] Brennan B., Kirton L., Marec-Berard P., Gaspar N., Laurence V., Martin-Broto J., Sastre A., Gelderblom H., Owens C., Fenwick N. (2022). Comparison of two chemotherapy regimens in patients with newly diagnosed ewing sarcoma (ee2012): An open-label, randomised, phase 3 trial. Lancet.

[32] Lindner L.H., Blay J.Y., Eggermont A.M.M., Issels R.D. (2021). Perioperative chemotherapy and regional hyperthermia for high-risk adult-type soft tissue sarcomas. Eur. J. Cancer.

[33] Issels R.D., Lindner L.H., Verweij J., Wessalowski R., Reichardt P., Wust P., Ghadjar P., Hohenberger P., Angele M., Salat C. (2018). Effect of neoadjuvant chemotherapy plus regional hyperthermia on long-term outcomes among patients with localized high-risk soft tissue sarcoma: The eortc 62961-esho 95 randomized clinical trial. JAMA Oncol..

[34] Fleuren E.D.G., Vlenterie M., van der Graaf W.T.A. (2023). Recent advances on anti-angiogenic multi-receptor tyrosine kinase inhibitors in osteosarcoma and ewing sarcoma. Front. Oncol..

[35] Khalifa J., Ouali M., Chaltiel L., Le Guellec S., Le Cesne A., Blay J.Y., Cousin P., Chaigneau L., Bompas E., Piperno-Neumann S. (2015). Efficacy of trabectedin in malignant solitary fibrous tumors: A retrospective analysis from the french sarcoma group. BMC Cancer.

[36] Chew H.Y., Chan V., Simpson F., Dolcetti R. (2020). Will next-generation immunotherapy overcome the intrinsic diversity and low immunogenicity of sarcomas to improve clinical benefit?. Cancers.

