# Supplementary material for: Prognostic Factors and Survival Outcomes in Resectable Thoracic Soft Tissue and Bone Sarcomas
Source: Cancers (Basel). 2026 Jun 11;18(12):1904. doi: 10.3390/cancers18121904 (PMC13297556; doi:10.3390/cancers18121904)
Supplement: Supplementary file 1 [file cancers-18-01904-s001.zip › cancers-4343910-supplementary.pdf]

Supplementary Table S1: Tumor Characteristics and Intraoperative Findings in Patients with Incomplete (R2) Resection

Patient	Age	Diagnosis	Tumor location	Operation	Intraoperative findings	Multimodal treatment	Outcomes
1	36	Osteosarcoma	sternal with multiple lung metastases	subtotal sternum resection and reconstruction with mesh, multiple wedge resections of the lung	Multiple pericardial and pleural metastases	neoadjuvant chemotherapy, BOSS-96 protocol	died 41 months later due to progressive metastatic disease
2	53	Angiosarcoma	intrathoracic infiltrating the diaphragm, liver, and pericardium	partial diaphragm resection, liver segmentectomy and partial pericardial resection with reconstruction	extensive infiltration of the pericardium	1 cycle adjuvant chemotherapy, AI 60/9	died 38 days later due to arrythmia caused by pericardial tumor infiltration
3	63	Liposarcoma	left thoracic and mediastinal infiltrating the esophagus, diaphragm, retroperitoneum, and aorta adventitia	resection of a mediastinal mass with wedge resection of lung, partial diaphragm resection, and mesh reconstruction	extensive infiltration of the aortic adventitia and esophagus	4 cycles adjuvant chemotherapy combined with hyperthermia	died 14 months later due to progressive disease
4	74	Undifferentiated pleomorphic sarcoma	intrathoracic pleural	decortication and pleurectomy	extensive tumor infiltration of the pleura	adjuvant trabectedin	died 2.8 months later due to progressive disease
5	52	Malignant triton tumor	chest wall	chest wall resection of the 3.-7. ribs with reconstruction using mesh and muscle flap	pleural metastases	4 cycles adjuvant chemotherapy and radiation	died 2.6 months later due to multiple bone metastases
6	35	Epithelioid hemangioendo-thelioma	intrathoracic infiltrating the diaphragm, pericardium and left upper lobe	decortication and pleurectomy with wedge resection of the left upper lobe	pleural and pericardial metastases	adjuvant pazopanib	died 5.5 months later due to progressive disease and ileus
